# Association of scopophobia with online learning fatigue among medical students in Brazil

**DOI:** 10.1186/s12909-023-04199-z

**Published:** 2023-04-06

**Authors:** Mateus Sudário Alencar, Marcos Kubrusly, Claudia Maria Costa de Oliveira, Bianca Oriá Almada de Aquino, Isadora Néri Viana, Pedro Iughetti Morais, Hermano Alexandre Lima Rocha

**Affiliations:** 1 Unichristus University Center, R. João Adolfo Gurgel, 133 - Cocó, Fortaleza, CE 60190-180 Brazil; 2 Department of Global Health and Population, Harvard T. H. Chan School of Public Health, Boston, MA USA

**Keywords:** Education, Medical, Fatigue, COVID-19, Education, Medical, Undergraduate

## Abstract

**Background:**

Scopophobia can be described in the medical field as the fear of being watched or stared at. Despite the relevance of scopophobia in remote learning scenarios, which have always existed and have been largely expanded during the pandemic in medical education, studies on this topic are exceedingly rare worldwide. Hence, to fill up this gap, a cross-sectional study of medical students was developed to assess the association of scopophobia with the prevalence of online learning fatigue.

**Methods:**

A cross-sectional, quantitative, analytical study was carried out in Medical Schools of Brazil. To assess the risk of scopophobia, questions were developed, based on the literature on the topic. The Zoom Exhaustion & Fatigue Scale (ZEF) was used, and the questions have currently been validated for Brazilian Portuguese. Logistic regression models were also used to assess the relationship of scopophobia risk and ZEF scores.

**Results:**

A total of 283 students from Brazil participated in the study. The median age was 23 years, and 64% of the participants were female. In total, 14.5% were considered to be at high risk for scopophobia. It was found that after adjusting for sex, income and number of residents in the household, scopophobia and the total zoom fatigue score remained associated. For the total score, each additional point on the scale increased the chance of scopophobia by 3%, and for the overall domain, 19% (p-values < 0.05).

**Conclusions:**

In conclusion, this study shows a relevant prevalence of students with scopophobia, which requires a differentiated approach on the part of teachers. The causes of scopophobia are often specific and have a psychological origin that goes beyond the usual pedagogical management. Therefore, motivation strategies are necessary in a general, as well as an individualized manner, aiming to favor the improvement of the online teaching and learning process.

**Supplementary Information:**

The online version contains supplementary material available at 10.1186/s12909-023-04199-z.

## Introduction

Scopophobia can be described, in the medical field, as the fear of being observed or stared at, mainly by unknown people. It occurs more commonly in women than in men, in addition to being often identified among young individuals. It may also be related to the fear of surveillance or of being manipulated by someone [1–3]. This phenomenon is present in academic environments, since with the current COVID-19 pandemic, it was necessary to change in-person teaching to the online modality, with the consequent increase in videoconferences [4, 5].

E-learning is the use of technology to facilitate learning beyond the traditional classroom setting. There are two main types of e-learning: synchronous and asynchronous. Synchronous e-learning is when students and instructors engage in learning activities simultaneously but in different locations, through live video conferencing, webinars or chat sessions. This type of e-learning allows for real-time interaction and feedback between students and instructors, providing a sense of community and social presence. Asynchronous e-learning, on the other hand, involves students accessing pre-recorded lectures, assignments, and quizzes at their own pace and time, with little or no live interaction with the instructor or other students [6]. Due to health restrictions during the COVID-19 pandemic, many classes and meetings migrated from in-person to the online modality, through videoconference platforms and interactive websites, including Zoom ©2023 Zoom Video Communications, Inc., which was chosen by many professionals due to its free and easy access. Soon, many people started using the word “zoom” when they referred to the action of making a video call. In Brazil, the synchronous mode of e-learning was mainly used, considering the guidelines of the Brazilian Ministry of Education for computing the students’ workload.

This abrupt transition from in-person to digital interactions raised discussions about the importance of “Zoom fatigue”, which refers to the feeling of exhaustion related to videoconferences after prolonged screen use and is associated with greater intellectual demand that is required in interpersonal relationships during video conferences, as non-verbal language becomes more difficult to perceive [7]. Therefore, “Zoom fatigue” has become frequent among students due to the increased use of remote interactive technologies. Students and teachers experience mental and physical fatigue, which impairs their learning abilities, lowers their motivation, and increases anxiety [8]. Additionally, the fear of consequences such as missing social interaction and decreased learning effectiveness can also negatively impact student performance [9]. Addressing and mitigating Zoom fatigue is vital for maintaining the effectiveness of online education.

Despite the relevance of scopophobia in the remote learning processes, which have always existed and increased exponentially in medical education during the pandemic, studies on this topic in the world are exceedingly rare. Hence, to fill up this gap, a cross-sectional study of medical students from different institutions from all over Brazil was developed to assess the association of scopophobia and the prevalence of online learning fatigue.

## Methods

### Study design

A cross-sectional, quantitative, analytical study was carried out in Medical Schools of Brazil. Social distancing due to the COVID-19 pandemic started in March 2020 in Brazil, when recommendations for maintaining remote medical education began. Despite the partial reopening of schools that took place at the end of 2020, the second wave of the pandemic hit Brazil at the end of 2020 with much more intensity, and all teaching activities were reverted to remote, with in-person activities only restarting in March 2022. The data collection period went from July 2021 to October 2021.

### Study population and sample

The study recruited individuals aged 18 years of age and older, of both genders, who attended higher education institutions in Brazil and were medical students. Students who did not use virtual platforms during the pandemic were excluded.

### Data collection

The data were collected using electronic Google forms © Alphabet, Inc. sent to Brazilian Medical Schools, which redirected them to the students.

### Variables

Considering the lack, to the best of our knowledge, of a tool to verify the increased risk of scopophobia, we developed questions, based on the literature about the subject, to assess a greater propensity of students to be affected by scopophobia [1–3, 10]. The students were asked to read the items and, based on their recent online classes, answer the questions using a Likert scale ranging from 1 to 5, where 1 meant “I strongly disagree” and 5 “I strongly agree”. The evaluated items are available in supplementary box 1.

The final variable was constructed as follows: if the student answered by using the maximal negative response on the Likert scale (“I strongly agree”), they would score one point in that item. If at the end the student attained 3 or 4 points in the 4 items, they would be categorized as being at high risk of having scopophobia.

These questions were defined after an extensive literature review that included studies since 1975 that studied the phenomenon and proposed explanations for its emergence, such as Becker’s [11] and Bailenson’s theories [12]. It should be noted that research about scopophobia is very rare. These items were then evaluated by two judges (psychiatrists) to validate the questions. We calculated the intraclass correlation coefficient (ICC) of the instrument, which is a descriptive statistic that describes how strongly units in the same group resemble each other, and we found the Cronbach`s alpha of 0.624, which is classified as a good result [13].

The English version of the Zoom Exhaustion & Fatigue Scale (ZEF) [14] was used in the study, with the questions being currently validated for Brazilian Portuguese [15]. This scale consists in a set of fifteen questions, divided into five domains: overall, visual, social, motivational and emotional, and assesses possible psychological damage occurring in each of these domains in participants of online interactions, both didactic and business ones (Cronbach`s alpha of 0.95). These domains are defined as: Overall fatigue refers to the superordinate experience of being tired (e.g., feeling drained); reduced motivation refers to a lack of motivation to start an activity (e.g., dread having to do things); visual fatigue is defined by the National Research Council Committee on Vision as “any subjective visual symptom or distress resulting from use of one’s eyes” and is measured with items such as “my vision seems blurry”; emotional fatigue, defined as “the state of feeling overwhelmed, drained and used up”, occurs after interactions with other people and includes items based on emotional symptoms related to fatigue, such as moodiness and irritability; social fatigue refers to feelings of wanting to be alone, which is derived from the interview and researchers’ experiences [16]. In the original article, ‘Zoom Fatigue’ was defined as the fatigue that can be experienced during or after participating in a videoconference. The variables were used continuously, as instructed by the scale developers. A self-reported questionnaire on sociodemographic data and life habits was also applied.

### Statistical analysis

Initially, the descriptive measures of the collected variables were presented, using frequencies and percentages for categorical variables and means and standard deviations for the numerical ones. The chi-square tests were used to verify the statistical association between the measured variables and scopophobia. Specifically for the variable semester in which the student is, we use the test Goodman and Kruskal’s lambda coefficient. Logistic regression models were also used to verify the occurrence of confounding factors among the variables identified as statistically associated with the outcome in the bivariate analysis. Values of p < 0.05 were considered statistically significant. Data were tabulated and statistical calculations were performed using the software Statistical Package for Social Sciences (SPSS), version 23.0 (SPSS Inc., Chicago, United States)^®^.

### Ethical aspects

In the online application, the Free and Informed Consent form was applied through the electronic platform and made available to all participants. All necessary procedures were adopted to keep the collected data confidential. The project was submitted to the Research Ethics Committee (REC) of Unichristus University.

## Results

A summary of the baseline characteristics of the study participants, which included 283 medical students, is shown in Table 1. The median age was 23 years, and 64% of the participants were female. Most participants were attending the eighth semester, and the majority were attending from the fourth to the eighth semesters. Additionally, most of the participating students were from the Northeast of Brazil. The family income of a little over three quarters of the participants was greater than five minimum wages, and most students lived with their parents, with 23% reporting the presence of children in the households. At the time of the study, more than 85% of the participants had been dealing with remote classes for more than a year.

**Table 1:** Description of the sample of evaluated medical students

	Total (N = 283)
**Age**	
N	283
Median (IQR)	23.0 (21.0, 26.0)
**Sex**, n (%)	
Female	181 (64.0%)
Male	100 (35.3%)
Other	1 (0.4%)
I would rather not answer it	1 (0.4%)
**Semester attending**, n (%)	
1st	1 (0.4%)
2nd	16 (5.7%)
3rd	4 (1.4%)
4th	50 (17.7%)
5th	50 (17.7%)
6th	26 (9.2%)
7th	30 (10.6%)
8th	94 (33.2%)
9th	8 (2.8%)
10th	3 (1.1%)
12th	1 (0.4%)
**Region of Brazil**, n (%)	
Northeast	252 (91.3%)
Southeast	11 (4.0%)
South	13 (4.7%)
Missing information	7
**Occupation**, n (%)	
Others	1 (0.4%)
I only study	246 (86.9%)
I work and study	36 (12.7%)
**Family income**, n (%)	
Up to 1 MW	10 (3.5%)
UP to 3 MWs	31 (11.0%)
Up to 5 MWs	25 (8.8%)
More than 5 MWs	217 (76.7%)
**Number of people in the same dwelling**, n (%)	
I live alone With 1 person	20 (7.1%) 49 (17.3%)
With 2 to 4 people	174 (61.5%)
With 5 or more people	40 (14.1%)
**Person the student lives with**, n (%)	
Parents	190 (67.1%)
Partner	41 (14.5%)
Alone	20 (7.1%)
Other	32 (11.3%)
**Existence of children in the dwelling** n (%)	
No	216 (76.3%)
Yes	67 (23.7%)
**Self evaluation of academic performance**, n (%)	
Better than usual	74 (26.1%)
The same as usual	71 (25.1%)
Worse than usual	138 (48.8%)
**Duration of participation in remote classes**, n (%)	
6 months	16 (5.7%)
1 year	25 (8.8%)
More than one year	242 (85.5%)

MW = minimum wage

The medical students’ perceptions about the use of cameras in remote teaching are shown in Table 2. About half of the respondents reported turning on their cameras during online classes, and 83.9% stated that they did so because it was mandatory, rather than because they thought it was important. On a scale of 0 to 10, students rated with a median score of 5 the importance of turning on the cameras during class, and of 4 how comfortable they felt when leaving the cameras on during class. Among the characteristics associated with scopophobia, 30% agreed that they felt like they were missing information when compared to if they were attending an in-person class; 50.8% thought they were looking into a mirror; 34.6% disagreed they felt closer to other participants and 50.9% agreed that they felt like they were being watched when the cameras were turned on. In total, 14.5% were considered to be at high risk for scopophobia. (Table 2)

**Table 2:** Medical students’ impressions on the use of cameras during remote teaching

	Total (N = 283)
**If yes, did you turn on your camera during online classes last semester?**, n (%)	
No	144 (50.9%)
Yes	139 (49.1%)
**If yes, did you turn it on because it was mandatory or do you think it is important to turn on the camera during online classes?** ,n (%)	
I thought it was important	36 (16.1%)
Because it was mandatory	188 (83.9%)
Missing information	59
**On a scale of 0 to 10, where 0 is little and 10 is a lot, how important did you think it was to turn on your camera during online classes last semester?**	
N	283
Median (IQR)	5.0 (2.0, 7.0)
**On a scale of 0 to 10, where 0 is little and 10 is a lot, how comfortable were you with turning on your camera during online classes last semester?**	
N	283
Median (IQR)	4.0 (1.0, 7.0)
**I thought the class was more productive when everyone had the camera on.**, n (%)	
I strongly disagree	114 (40.3%)
I disagree	46 (16.3%)
I neither agree, not disagree	40 (14.1%)
I agree	41 (14.5%)
I strongly agree	42 (14.8%)
**I was able to concentrate more on class when the camera was on.**, n (%)	
I strongly disagree	102 (36.0%)
I disagree	36 (12.7%)
I neither agree, not disagree	53 (18.7%)
I agree	39 (13.8%)
I strongly agree	53 (18.7%)
**How often do you participate in video conferences, on average?**, n (%)	
Never	17 (6.0%)
Once a month	45 (15.9%)
Once a week	107 (37.8%)
Once a day	40 (14.1%)
Several times a day	74 (26.1%)
**With the cameras on, I had the illusion of being close and actually having little information about what was going on, compared to what I would have felt with in-person classes.**, n (%)	
I strongly disagree	85 (30.0%)
I disagree	44 (15.5%)
I neither agree, not disagree	69 (24.4%)
I agree	42 (14.8%)
I strongly agree	43 (15.2%)
**When the camera was on, it gave me the impression that I was constantly looking into a mirror.**, n (%)	
I strongly disagree	63 (22.3%)
I disagree	38 (13.4%)
I neither agree, not disagree	38 (13.4%)
I agree	59 (20.8%)
I strongly agree	85 (30.0%)
**When the camera was on, it made me feel like I was closer and more exposed to the other participants in the class than I would like to be.**, n (%)	
I strongly disagree	55 (19.4%)
I disagree	43 (15.2%)
I neither agree, not disagree	46 (16.3%)
I agree	66 (23.3%)
I strongly agree	73 (25.8%)
**When the camera was on, it made me feel like I was being watched and that everyone was looking at me.**, n (%)	
I strongly disagree	55 (19.4%)
I disagree	35 (12.4%)
I neither agree, not disagree	49 (17.3%)
I agree	47 (16.6%)
I strongly agree	97 (34.3%)
**High risk of scopophobia**, n (%)	
No	242 (85.5%)
Yes	41 (14.5%)

**Fig. 1:**
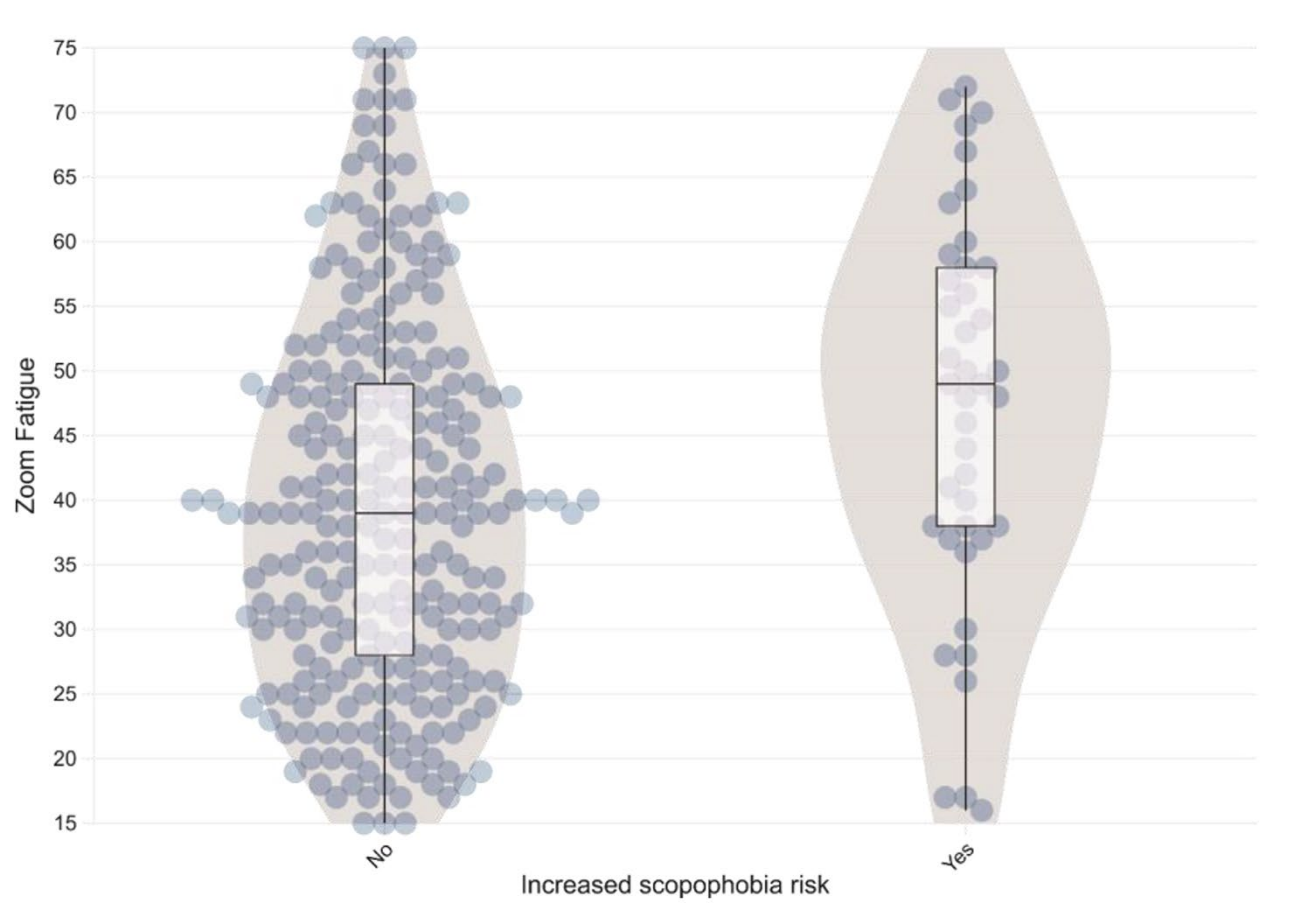
Violin plot of the distribution of the total score of the zoom fatigue scale according to the presence of high risk of scopophobia

When studying the factors associated with a high risk of scopophobia, we can observe that females have a higher prevalence of high risk, as seen in Table 3. Also, having a lower family income and living alone were also associated with a higher occurrence of scopophobia. The total zoom fatigue score, as shown in Fig. 1, as well as all its domains, were statistically associated with scopophobia, with higher scores being verified in positive cases.

**Table 3:** Factors associated with scopophobia in the assessed sample

	High risk of scopophobia			
	No (N = 242)	Yes (N = 41)	Total (N = 283)	p-value
**Age**				0.9140^1^
N	242	41	283	
Median (IQR)	22.0 (21.0, 26.0)	23.0 (21.0, 25.0)	23.0 (21.0, 26.0)	
**Sex**, n (%)				0.0231^2^
Female	150 (62.0%)	31 (75.6%)	181 (64.0%)	
Male	91 (37.6%)	9 (22.0%)	100 (35.3%)	
Other	1 (0.4%)	0 (0.0%)	1 (0.4%)	
I would rather not answer it	0 (0.0%)	1 (2.4%)	1 (0.4%)	
**Semester attending**, n (%)				0.3164^3^
1st	1 (0.4%)	0 (0.0%)	1 (0.4%)	
2nd	12 (5.0%)	4 (9.8%)	16 (5.7%)	
3rd	2 (0.8%)	2 (4.9%)	4 (1.4%)	
4th	43 (17.8%)	7 (17.1%)	50 (17.7%)	
5th	46 (19.0%)	4 (9.8%)	50 (17.7%)	
6th	19 (7.9%)	7 (17.1%)	26 (9.2%)	
7th	24 (9.9%)	6 (14.6%)	30 (10.6%)	
8th	85 (35.1%)	9 (22.0%)	94 (33.2%)	
9th	7 (2.9%)	1 (2.4%)	8 (2.8%)	
10th	3 (1.2%)	0 (0.0%)	3 (1.1%)	
12th	0 (0.0%)	1 (2.4%)	1 (0.4%)	
**Region of Brazil**, n (%)				0.2663^2^
Northeast	219 (92.4%)	33 (84.6%)	252 (91.3%)	
Southeast	8 (3.4%)	3 (7.7%)	11 (4.0%)	
South	10 (4.2%)	3 (7.7%)	13 (4.7%)	
Missing information	5	2	7	
**Occupation**, n (%)				0.4836^2^
Others	1 (0.4%)	0 (0.0%)	1 (0.4%)	
I only study	208 (86.0%)	38 (92.7%)	246 (86.9%)	
I work and study	33 (13.6%)	3 (7.3%)	36 (12.7%)	
**Family income**, n (%)				0.0182^2^
Up to 1 MW	6 (2.5%)	4 (9.8%)	10 (3.5%)	
Up to 3 MWs	23 (9.5%)	8 (19.5%)	31 (11.0%)	
Up to 5 MWs	21 (8.7%)	4 (9.8%)	25 (8.8%)	
More than 5 MWs	192 (79.3%)	25 (61.0%)	217 (76.7%)	
**Number of people in the same dwelling**, n (%)				0.0011^2^
With 1 person	40 (16.5%)	9 (22.0%)	49 (17.3%)	
With 2 to 4 people	158 (65.3%)	16 (39.0%)	174 (61.5%)	
With 5 or more people	32 (13.2%)	8 (19.5%)	40 (14.1%)	
I live alone	12 (5.0%)	8 (19.5%)	20 (7.1%)	
**Person the student lives with**, n (%)				0.0618^2^
Partner	36 (14.9%)	5 (12.2%)	41 (14.5%)	
Other	28 (11.6%)	4 (9.8%)	32 (11.3%)	
Parent(s)	165 (68.2%)	25 (61.0%)	190 (67.1%)	
Alone	13 (5.4%)	7 (17.1%)	20 (7.1%)	
**Existence of children in the dwelling**, n (%)				0.9072^2^
No	185 (76.4%)	31 (75.6%)	216 (76.3%)	
Yes	57 (23.6%)	10 (24.4%)	67 (23.7%)	
**Self evaluation of academic performance**, n (%)				0.7528^2^
The same as usual	61 (25.2%)	10 (24.4%)	71 (25.1%)	
Better than usual	65 (26.9%)	9 (22.0%)	74 (26.1%)	
Worse than usual	116 (47.9%)	22 (53.7%)	138 (48.8%)	
**ZEF, total score**				0.0023^1^
N	242	41	283	
Median (IQR)	39.0 (28.0, 49.0)	49.0 (38.0, 58.0)	40.0 (29.0, 50.0)	
Mean (SD)	39.5 (14.4)	47.0 (14.9)	40.5 (14.7)	
Min - Max	15–75	16–72	15–75	
**ZEF, overall score**				0.0004^1^
N	242	41	283	
Median (IQR)	9.0 (7.0, 12.0)	12.0 (9.0, 14.0)	9.0 (7.0, 12.0)	
Mean (SD)	9.2 (3.2)	11.1 (3.4)	9.4 (3.3)	
Min - Max	3–15	3–15	3–15	
**ZEF, visual score**				0.0081^1^
N	242	41	283	
Median (IQR)	6.0 (4.0, 9.0)	9.0 (6.0, 10.0)	6.0 (5.0, 9.0)	
Mean (SD)	7.1 (3.4)	8.4 (3.1)	7.2 (3.4)	
Min - Max	3–15	3–15	3–15	
**ZEF, social score**				0.0280^1^
N	242	41	283	
Median (IQR)	6.0 (4.0, 9.0)	8.0 (6.0, 12.0)	6.0 (4.0, 10.0)	
Mean (SD)	7.0 (3.5)	8.2 (3.6)	7.1 (3.7)	
Min - Max	3–15	3–14	3–15	
**ZEF, motivational score**				0.0152^1^
N	242	41	283	
Median (IQR)	9.0 (7.0, 12.0)	11.0 (8.0, 13.0)	9.0 (7.0, 12.0)	
Mean (SD)	9.2 (3.3)	10.5 (3.7)	9.4 (3.4)	
Min - Max	3–15	3–15	3–15	
**ZEF, emotional score**				0.0026^1^
N	242	41	283	
Median (IQR)	7.0 (4.0, 9.0)	10.0 (6.0, 11.0)	7.0 (5.0, 10.0)	
Mean (SD)	7.1 (3.2)	8.8 (3.5)	7.4 (3.3)	
Min - Max	3–15	3–15	3–15	

^1^Kruskal-Wallis p-value; ^2^Chi-Square p-value, ^3^Goodman and Kruskal’s lambda coefficient p-value; MW = minimum wage

**Fig. 2:**
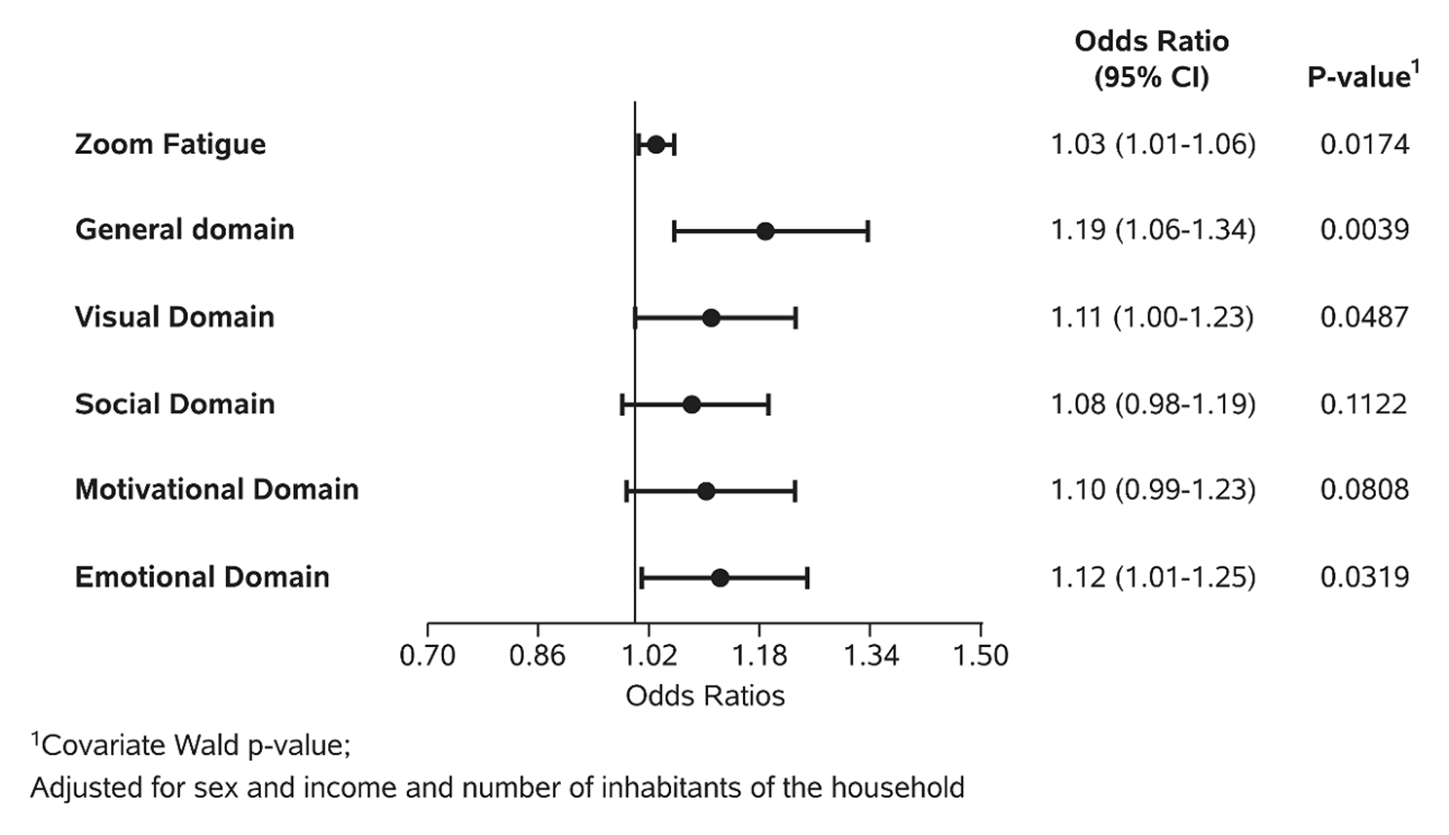
Forest plot of the adjusted odds ratios of the association of scores of the zoom fatigue scale and the presence of high risk of scopophobia

After the bivariate analysis, the factors identified as associated with scopophobia were used to construct the multivariate model, shown in Fig. 2. It was found that after adjusting for sex, income and number of residents in the household, the total zoom fatigue score and the scores of the overall, visual and emotional domains remained associated with scopophobia, with statistical significance. For the total score, each additional point on the scale increased the chance of scopophobia by 3%, and for the overall score, 19% (Fig. 2).

## Discussion

In this study carried out during the period of social distancing due to the COVID-19 pandemic, it was observed that the prevalence of a high risk of scopophobia among medical students is high, being associated with the prevalence of zoom fatigue, with a higher prevalence being identified in students with higher scores of zoom fatigue.

The use of cameras during a synchronous class represents a great challenge in hybrid learning, as well as in remote learning that was intensely experienced during the COVID-19 pandemic. The present study showed that approximately 50% of the students turned on the cameras during synchronous classes and had a reasonable perception of its importance and comfort when using them. One of the reasons for choosing to keep the camera on may be found in the study by Živilė Sederevičiūtė-Pačiauskienė et al., where students experienced social distancing when their peers turned off the camera and felt less likely to participate when not using their video cameras. Therefore, the students associated the use of the video cameras with the community, integration and cooperative assistance [1].

The students understood that, without the cameras, they lose their relationships with teachers and classmates; in that case, the social interaction would be absent and “social learning would not occur” [17]. However, 34.6% of the students analyzed in the present study disagree with the fact of feeling closer to other participants in online classes when they have the camera on.

Other studies corroborating the same reasoning have shown that student-teacher relationships during video learning are crucial for academic success and student satisfaction [18, 19]. Along the same line of thought, Garrison et al. (1999) adapting Dewey’s philosophy, said that three central elements must be present in the online environment to facilitate learning: a social presence, a cognitive presence and a teacher presence [20]. In addition, when the cameras are on, they are positively implicated in favoring non-verbal communication in the virtual learning environment. This body cue through facial expressions plays an essential role, since much of human communication occurs through non-verbal communication (body language) and the latter must always be synchronized with verbal communication to attain its full function [21]. With regard to learning through active methodologies, Kubrusly et al. showed that during the online tutorial session, most tutors agree with the need to leave the cameras on during the sessions, at the risk of negatively affecting the tutor-student interaction and, consequently, the formative assessment of the tutorial session [22]. Despite the described justifications, Bradner and Mark (2001) showed that visual feedback from a collaborating partner (or observer) is not necessary to create a sense of presence [23].

On the other hand, we showed that more than 50% of the students did not turn on the cameras during the synchronous classes in the last semester, which is in disagreement with the results of FR Castelli and MA Sarvary (2020) with undergraduate students, who revealed several reasons why students do not turn on their video cameras; among the most important concerns were those about one’s personal appearance and other people’s opinions [4]. Furthermore, Nowak et al. observed that people prefer to perform a task using less effort than more effort [24]. If the students could participate in the synchronous remote learning classroom with an audio setup only, they would likely choose this option. Moreover, switching to online teaching was a baptism of fire for many students, as they lacked the experience and trust in online teaching and described the progress of online learning as a sort of “black box”, clearly frustrated by the lack of direct interaction and feedbacks [25].

In addition to the students’ preference to turn off the cameras, only 18.7% of the interviewees had a perception of a maximum concentration by keeping the cameras on. This low percentage of concentration can be explained by the increase in sustained attention of videoconferences, making them more exhausting than in-person sessions and due to the greater demand for focus than in-person classes [26]. This occurs because we have to work a little harder to process the body language as well as one’s tone of voice, which means “we cannot naturally relax into the conversation” [27]. Regarding the obligation to turn on the cameras, 83.9% of the students in our study declared they did it because it was mandatory, rather than because they thought it was important.

Overall, experts disagree on this obligation to turn on the camera and microphone during class. Some come together to state that schools can compel the students to do so, while others disagree, and understand that it would be a violation of the young people’s rights [28]. Castelli and Sarvary, 2021, proposed strategies to encourage – without demanding – the use of cameras, while promoting equity and inclusion [4]. By explaining to students the rationale behind recommending the use of the camera during synchronous class sessions, the instructor helps to set the standards for the course and maintains the transparency on how the camera use will improve the learning experience. Thus, our results show the need to increasingly strengthen the students’ motivation, the feeling of belonging during online activities. This can be achieved by encouraging students to use their cameras during synchronous remote classes and equally promoting interactive participation; this will be essential, especially for first-year students who are still developing virtual learning habits, a learning activity that was strengthened during the pandemic and which will remain in the pedagogical processes of the current higher educational institutions, being what we now call hybrid teaching. One also must pay attention to the cognitive overload that can result from a greater number of online activities, as well as the time spent using this activity and its methodology. In our study more than 40% of the students attended one or more online classes a day and more than 85% of the participants had been dealing with remote classes for more than a year. Kubrusly et al. showed in their study that the videoconference duration, as well as the type of teaching methodology used can be decisive for the onset of zoom fatigue [29].

The digital leap has spurred a global debate with education experts about the use of webcams in online classes. The refusal to keep the camera on by some of the students cannot be interpreted exclusively as favoring the students’ non-participation in classes, preventing them from answering questions and being justified by the lack of connectivity. Its cause is multifactorial, such as personality traits, contextual factors, including human, family and technological resources available to students [30]. The present study showed the percentages of some psychological impacts that were asked to students in relation to the use of cameras. About 51% of the respondents had the impression they were looking into a “mirror” during online classes. When the student looks at their self-view video, the video appears as if they are looking at their reflection in a mirror, leading to a state of self-awareness, i.e., the video is always on and showing your appearance [31]. It can also lead to a state of public self-awareness, where the student focuses attention on aspects of themselves that can be perceived by others. This level of concern that the student has with their appearance may be due to a number of different psychological and social factors that are beyond the teacher’s control [4]. The “spotlight effect”, i.e., the students’ feeling that they are being watched more than they really are, was perceived by 50.9% of the students, which represents a stress factor. This finding may also be related to the “information bias”, which is when people favor information that confirms beliefs they already had, thus processing the information in a more negative way and focusing on what confirms that belief [27].

The total score of zoom fatigue, as shown in Fig. 1, as well as all its domains, were statistically associated with scopophobia, which is to be expected since, among the causes of this syndrome are ; (1) the increased cognitive load due to the effort required to guess the non-verbal messages of others, whereas in real-life interactions, they flow naturally and effortlessly; (2) looking at one’s own face all day makes us more self-aware and more critical of our “self-awareness” appearance, and (3) there is a reduced capacity to move and gesture during online activities, which negatively affects the creativity and efficiency of a meeting. Additionally, online interactions are perceived as artificial; even with the cameras on, zoom fatigue is a problem for many individuals. Also, someone can be distracted by their own face and trying to look good and interested, which tends to affect one’s concentration [32].

This study has some limitations. First, as this is a cross-sectional study, associations that are not causal or show reverse causality can be observed. However, it is important to note that the two conditions can feed back into each other. Second, we used scales that screens scopophobia and zoom fatigue but are not diagnostic of clinical disorders. Despite these facts, the validity of the zoom fatigue scale has been demonstrated, and we were very conservative with the scopophobia scale and still found a high prevalence. Finally, the application of online questionnaires may have led to a non-random selection.

Thus, considering that online learning may persist for years beyond the COVID-19 pandemic, it is important to know and provide instructions on how to reduce scopophobia and the associated video conference fatigue. In conclusion, this study shows a significant prevalence of scopophobia among medical students, which supports the need for a differentiated approach by the teachers. The causes of scopophobia are often specific and have a psychological origin that goes beyond the usual pedagogical management. Therefore, motivation strategies are necessary in a general as well as individualized manner, aiming to improve the online teaching and learning process.

## Electronic supplementary material

Below is the link to the electronic supplementary material.

Supplementary Material 1

## Data Availability

The datasets used and/or analyzed during the current study are available from the corresponding author on reasonable request.

## List of Abbreviations

PBL: Problem-Based Learning
