# Supplementary material for: Association of scopophobia with online learning fatigue among medical students in Brazil
Source: BMC Med Educ. 2023 Apr 6;23:221. doi: 10.1186/s12909-023-04199-z (PMC10079143; doi:10.1186/s12909-023-04199-z)
Supplement: Supplementary file 1 — Supplementary Material 1 [file 12909_2023_4199_MOESM1_ESM.docx]

Supplementary box 1. Questions used to assess scopophobia among medical students.

- With the cameras on, I had the illusion of being close and actually having little information about what was going on, compared to what I would have felt with in-person classes.

- When the camera was on, it gave me the impression that I was constantly in front of a mirror.

- When the camera was on, it made me feel like I was closer and more exposed to the other participants in the class than I would like to be.

- When the camera was on, it made me feel like I was being watched and that everyone was looking at me.
